# Application of multiple injections of contrast agent in head and neck CT arteriovenous angiography in children: Study protocol clinical trial (SPIRIT Compliant)

**DOI:** 10.1097/MD.0000000000019773

**Published:** 2020-04-10

**Authors:** Shilong Tang, Guanping Zhang, Zhuo Chen, Xianfan Liu, Xiao Fan, Daisong Liu, Ling He

**Affiliations:** a Department of Radiology; b Ministry of Education Key Laboratory of Child Development and Disorders; c National Clinical Research Center for Child Health and Disorders; d China International Science and Technology Cooperation base of Child development and Critical Disorders; e Chongqing Key Laboratory of Pediatrics, Children's Hospital of Chongqing Medical University, Chongqing, China.

**Keywords:** angiography, children, contrast agent, head and neck, injection method of contrast agent

## Abstract

**Objective:**

To investigate the application value of multiple injections of contrast agent in head and neck CT arteriovenous angiography in children.

**Methods:**

A total of 100 children aged 6 to 7 years who needed head and neck CT arteriovenous angiography were prospectively selected. They were randomly divided into a control group and a research group, with 50 children in each group. The same scanning parameters and reconstruction methods were used. The right median cubital vein was injected intravenously with the contrast agent Omnipaque (350 mg I/ml). For children in the control group, a bolus of undiluted contrast agent (dose was 2 ml/kg, upper limit was 50 ml) was injected 1 time. The arterial phase and vein phase of the head and neck vessels were scanned. For children in the research group, a contrast agent bolus diluted with saline to a concentration of 20% was first injected (dose was 1 ml/kg, upper limit was 25 ml), and then an undiluted contrast agent bolus (dose was 1 ml/kg, upper limit was 25 ml) was injected. Thresholds were used to trigger the scanning of the head and neck arterial phases. The CT image quality of the head and neck arteries and veins, radiation dose and contrast agent dose were compared between the 2 groups.

**Results:**

Subjective evaluation of CT image quality of arteries: there were 47 cases of 4 points and 3 cases of 3 points in the control group and 34 cases of 4 points and 16 cases of 3 points in the research group. Subjective evaluation of CT image quality of veins: there were 47 cases of 4 points and 3 cases of 3 points in the control group and 5 cases of 4 points, 42 cases of 3 points and 3 cases of 2 points in the research group. The CT value of brain arterial vessel enhancement was higher in the control group than the research group, and the difference was statistically significant (*P* < .05). The CT value of vein enhancement was higher in the control group than the research group, and the difference was statistically significant (*P* < .05). The X-ray dose in the research group was 51% lower than that in the control group; the contrast agent dose in the research group was 44% lower than that in the control group.

**Conclusion:**

For the head and neck enhanced CT examination of children, the method of first bolus injection of 20% diluted contrast agent and later bolus injection of undiluted contrast agent can clearly demonstrate the head and neck arteries and veins one time, reducing the X-ray dose and contrast agent dose, which has clinical practical value in the enhanced CT examination of children with head and neck disease.

## 1 Introduction

With the increasing speed of CT imaging, the scope and frequency of CT examinations have increased in clinical practice, and enhanced CT examination has become an important way to screen head and neck vascular diseases. However, CT scans deliver a large dose of radiation, and the head and neck contain the thyroid, lens and other organs that are highly sensitive to X-irradiation. Children have significantly higher sensitivity to X-irradiation than adults, so reducing the radiation dose of CT scans for children is one of the responsibilities of medical staff.^1^,^2^,^3^,^4^,^5^,^6^ The contrast agent used in enhanced CT examination will increase the burden of renal filtration in children, and adverse reactions to contrast agents may occur, which are related to the dose and the injection rate of contrast agents. The development of kidneys in children is not perfect, and the kidneys capacity to filter contrast agents is lower than that of adults, so reducing the contrast agent dose in enhanced CT examination of children has become a hot spot of research for medical staff.^7^,^8^,^9^,^10^,^11^,^12^,^13^

In previous studies, radiologists often reduced the radiation dose to patients during CT examination by reducing the tube voltage and tube current, increasing the scanning screw pitch, and appropriately reducing the dose of contrast agent to reduce the burden of renal filtration and the incidence of adverse reactions of patients to contrast agent. However, the head and neck arteries and veins still need to be scanned twice, and the reduction of contrast agent dose is limited. Few studies have reduced the radiation dose and contrast agent dose of children by changing the injection method of the contrast agent bolus to reduce the damage of enhanced CT examination to children.^8^,^14^,^15^,^16^,^17^ This study explored the application value of changing the injection method of the contrast agent bolus to reduce the contrast agent dose and scanning radiation dose in enhanced CT examination of head and neck vessels in children.

## 2 Data and methods

The study protocol was approved by the Human Ethics Committee of the Children's Hospital of Chongqing Medical University. Written informed consent was obtained from the parents or guardians of all patients before the examinations.

## 3 Trial registration

### 3.1 General data

A total of 100 children aged 6 to 7 years who underwent enhanced CT of head and neck arteries and veins in our hospital from June 2018 to May 2019 were screened and collected. They were randomly divided into the control group and research group, with 50 children in each group. Control group: male to female ratio 24:26, mean age 6.5 ± 0.4 years, mean body weight 21.6 ± 2.1 kg; research group: male to female ratio 23:27, mean age 6.6 ± 0.3 years, mean body weight was 20.7 ± 1.9 kg.

### 3.2 Equipment and methods

**Figure 1:**
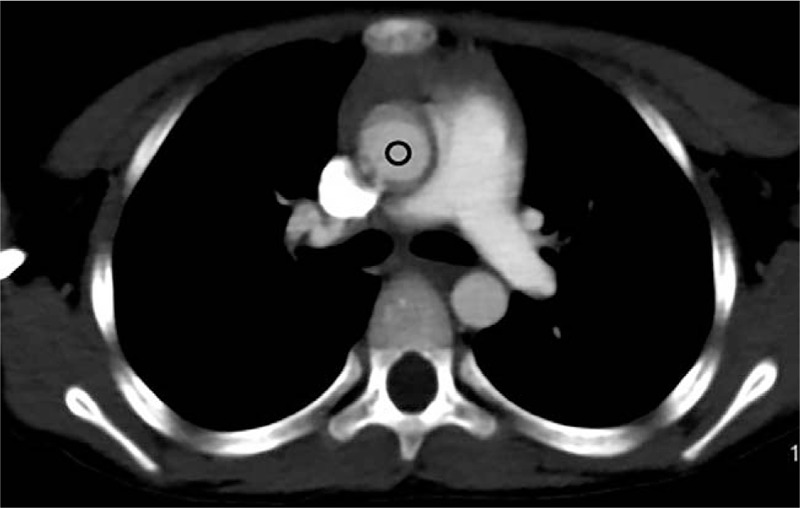
Schematic diagram of the scanning trigger threshold area of interest.

The children were scanned with a CT scanner (Philips Brilliance iCT, Philips, Netherlands), and children who did not cooperate were sedated. The scanning parameters of the 2 groups were consistent: 100 kV, 100 mAs, and screw pitch 0.4, and the idose^4^ iterative reconstruction algorithm was used for data processing. The scanning range was from calvaria to aortic arch, the scanning direction was from the foot side to the head side, the thickness of the scanning layer was 5 mm, and the thickness of the reconstruction layer was 1 mm. A threshold trigger was used for the scanning. The trigger zone was set at the center of the ascending aorta at the horizontal level below the tracheal carina, with a circular region of interest (ROI). The size of the ROI was 10 mm^2^, and the trigger threshold was 180 (Fig. 1). A double-barrel high-pressure syringe (Empower CTA, Bracco Injeneering S. A company, USA) was used, the contrast agent was Omnipaque (350 mg I/m), and the injection flow rate was 3 ml/second. The control group: an undiluted contrast agent bolus (dose was 2 ml/kg, upper limit was 50 ml) was injected into the right median cubital vein 1 time, and then the 2 phases, arterial and venous, of the head and neck were scanned. The research group: a contrast agent bolus diluted with saline to a concentration of 20% was first injected (dose was 1 ml/kg, the upper limit was 25 ml) into the right median cubital vein, and then the undiluted contrast agent bolus (dose was 1 ml/kg, the upper limit was 25 ml) was injected. The arterial phase of the head and neck was scanned.

#### 3.2.1 Data measurement

**Figure 2:**
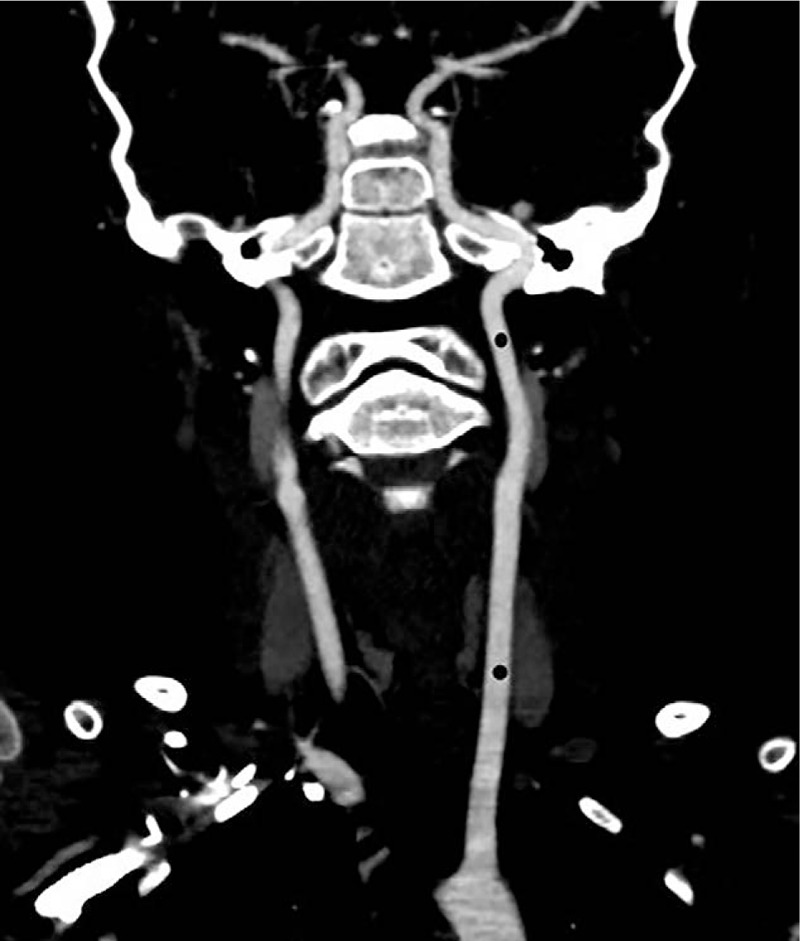
Schematic diagram of the measurement of the enhanced CT values of the common carotid artery and internal carotid artery.

**Figure 3:**
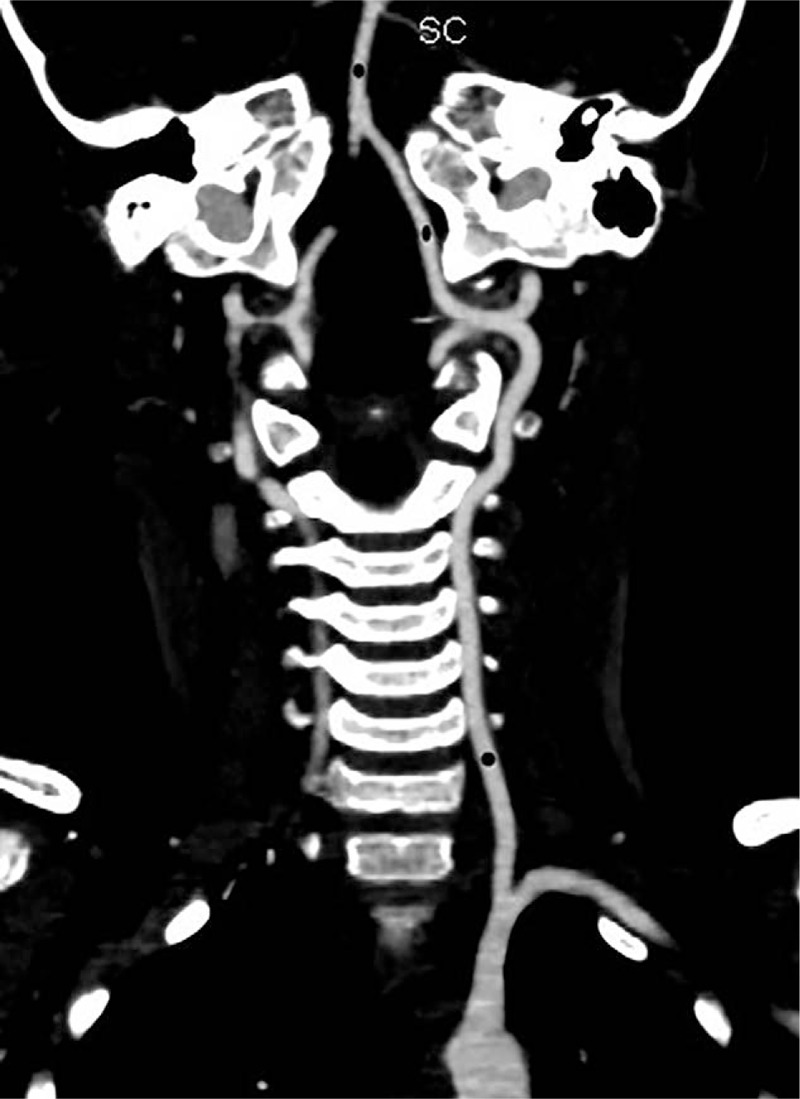
Schematic diagram of the measurement of the enhanced CT values of the vertebral artery, intracranial part of the vertebral artery and basilar artery.

**Figure 4:**
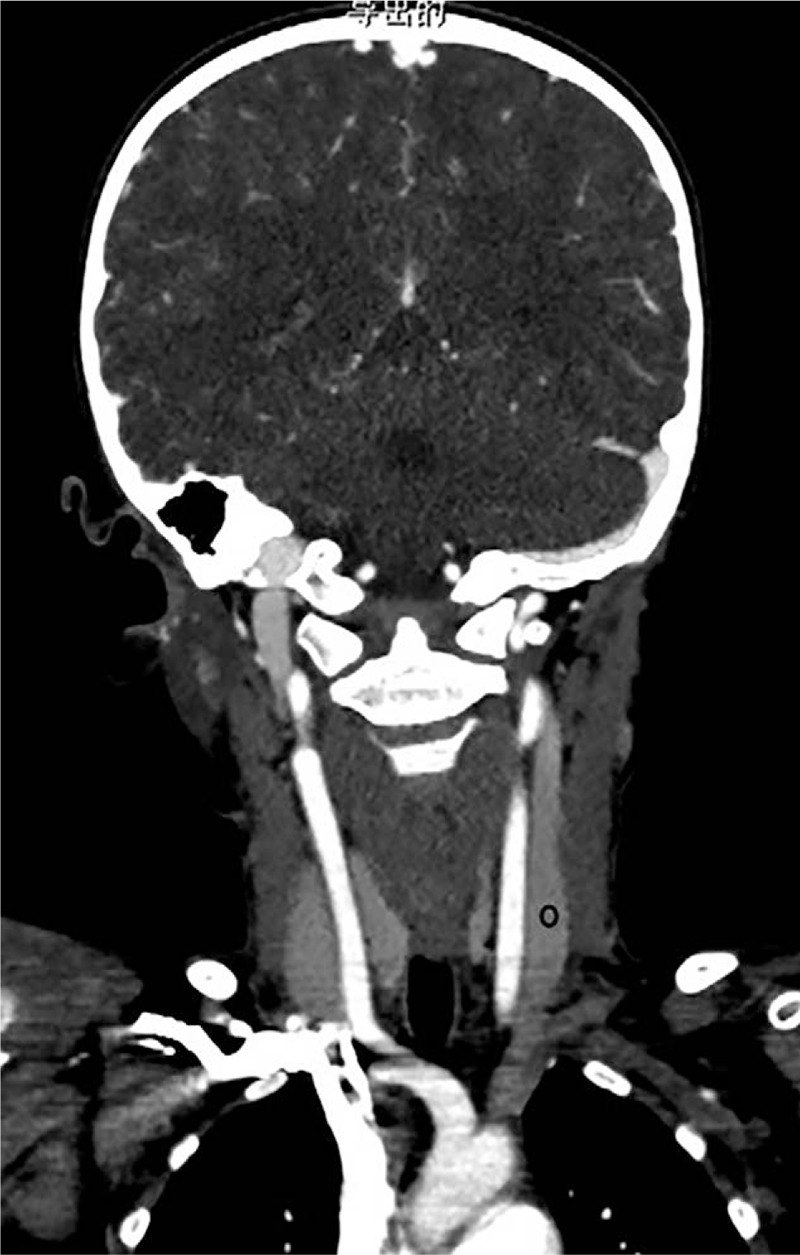
Schematic diagram of the measurement of the enhanced CT value of the jugular vein.

**Figure 5:**
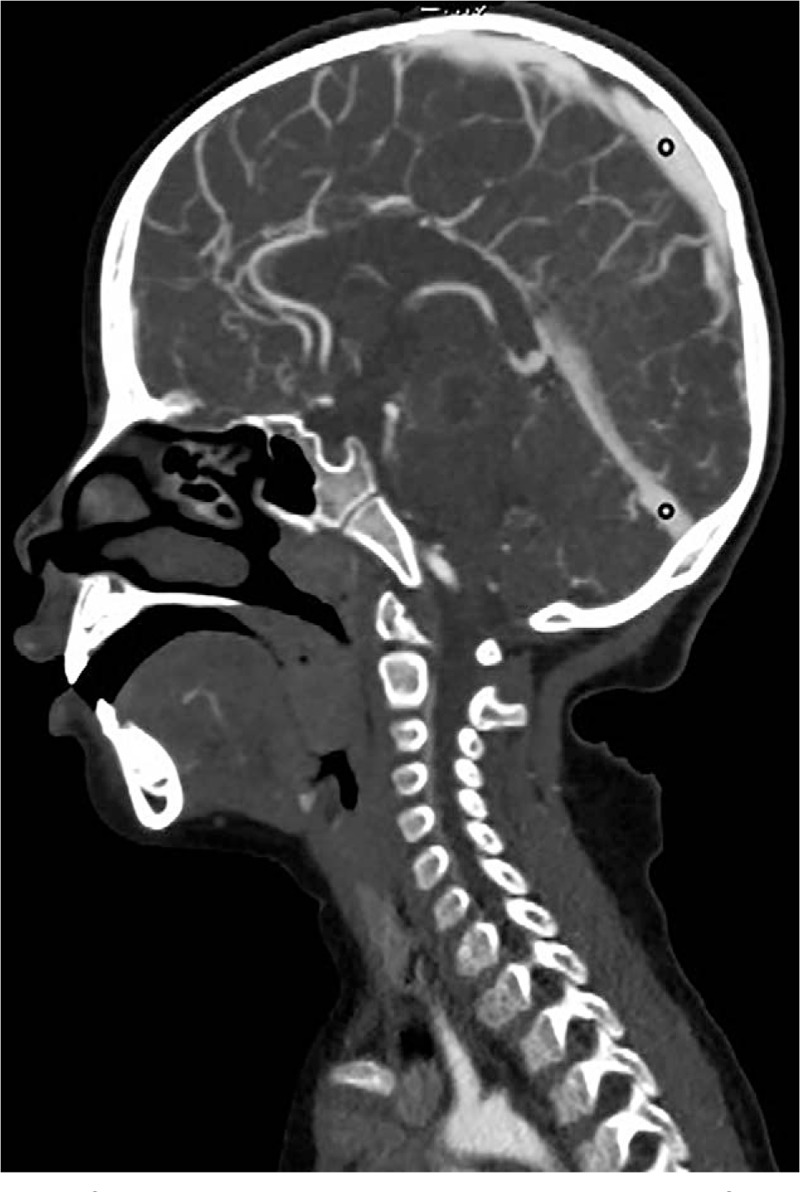
Schematic diagram of the measurement of the enhanced CT values of the superior sagittal sinus and straight sinus.

The IntelliSpace Portal V6.0 workstation of Philips was used. The raw data were imported into the postprocessing software to obtain the VR and MIP images of the head and neck arterial and venous vessels. The CT enhancement values of the common carotid artery, internal carotid artery, vertebral artery, intracranial part of vertebral artery, basilar artery, jugular vein, superior sagittal sinus, and straight sinus were measured. The positions of all children for measuring the vascular CT enhancement values were as consistent as possible.^18^,^19^,^20^,^21^,^22^ (Figs. 2–5). The above data were measured by a supervising radiology technician and 2 radiological technologists with 5 years of work experience.

#### 3.2.2 Radiation dose

The dose length product (DLP) value and CT dose index volume (CTDIvol) of the research group and the control group were recorded, and the effective dose (ED) value was calculated. The calculation formula was: ED = DLP × k, where the k value was taken as 0.0085 mSv · mGy^−1^ · cm^−1^.^[23]^ All the above data were taken as means.

#### 3.2.3 Subjective evaluation of vascular image quality

The 4-point system was used according to the vascular image quality of head and neck arteries and veins. Four points: the head and neck arteries and veins were well imaged with clear vascular boundaries and without artifacts, and any lesion could be clearly diagnosed; 3 points: the head and neck arteries and veins were relatively well imaged with relatively clear vascular boundaries and without artifacts, and imaging quality did not affect the clinical diagnosis; 2 points: the head and neck arteries were relatively well imaged and the veins were relatively poorly imaged, or the head and neck veins were relatively well imaged and the arteries were relatively poorly imaged, and imaging quality affected the clinical diagnosis; 1 point: both the head and neck arteries and veins were relatively poor imaged, and no lesion could be diagnosed.^24^,^25^,^26^,^27^,^28^ Subjective evaluation of the quality of vascular images was performed by 2 radiologists with more than 5 years of work experience. If there was any disagreement, it would be resolved by another radiologist with a senior title.

#### 3.2.4 Objective evaluation of image quality of arteries and veins

The enhanced CT values of arteries and veins were compared.

Statistical analysis: SPSS 22.0 statistical software was used; the measurement data are expressed as 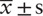. The vascular enhanced CT values at the same site in the research group and the control group were compared by paired T test (*P* < .05 was considered statistically significant). The X-ray dose and contrast agent dose of children and the subjective evaluation scores of arteries and veins in the research group and the control group were compared by paired T test (*P* < .05 was considered statistically significant).

## 4 Results

### 4.1 The objective evaluation of image quality of arteries and veins

The enhanced CT values of the arteries at each site: the research group had lower values than the control group (*P* < .05, the difference was statistically significant). The enhanced CT values of the veins at each site: the research group had lower values than the control group (*P* < .05, the difference was statistically significant) (Tables 1–2).

**Table 1:**
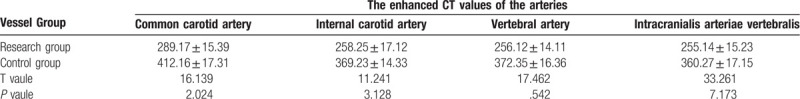
Objective evaluation results of image quality of arteries in children (n = 50, 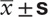).

**Table 2:**
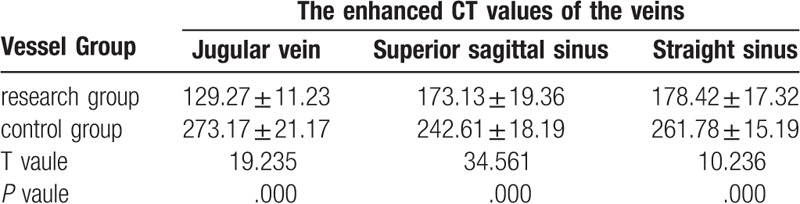
Objective evaluation results of image quality of veins in children (n = 50, 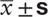).

### 4.2 The subjective evaluation of image quality of arteries and veins

**Figure 6:**
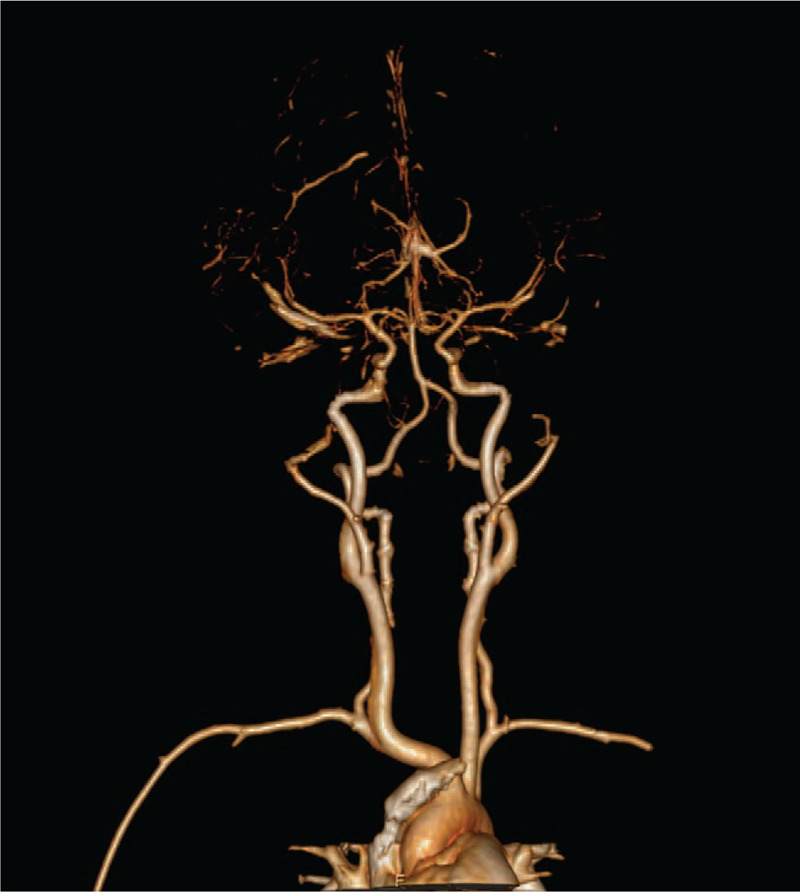
Research group: the same child, male, 6 years and 5 months, VR images of the head and neck vessels. It can be seen from the figure that the head and neck arteries, the superior sagittal sinus and the straight sinus are well imaged.

**Figure 7:**
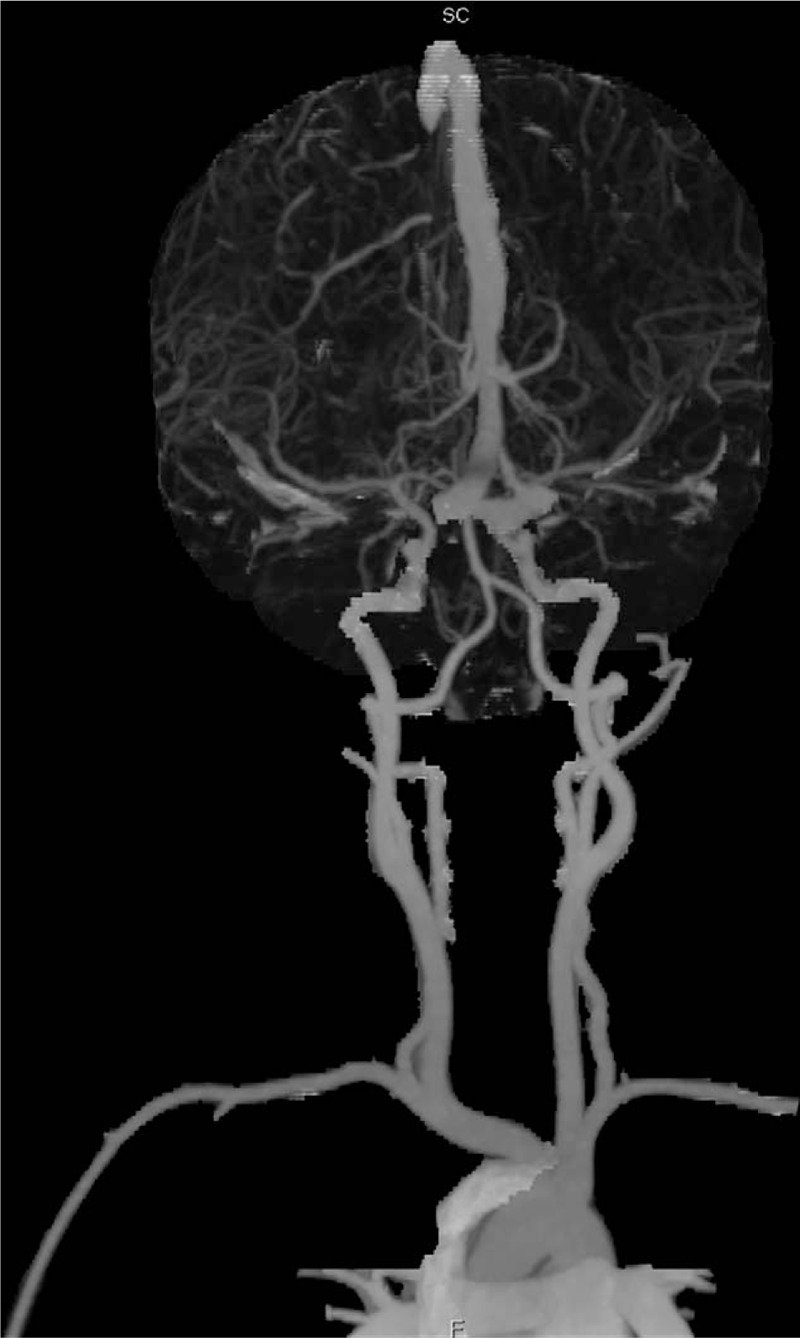
Research group: the same child, male, 6 years and 5 months, MIP images of the head and neck vessels. It can be seen from the figure that the head and neck arteries, the superior sagittal sinus and the straight sinus are well imaged.

**Figure 8:**
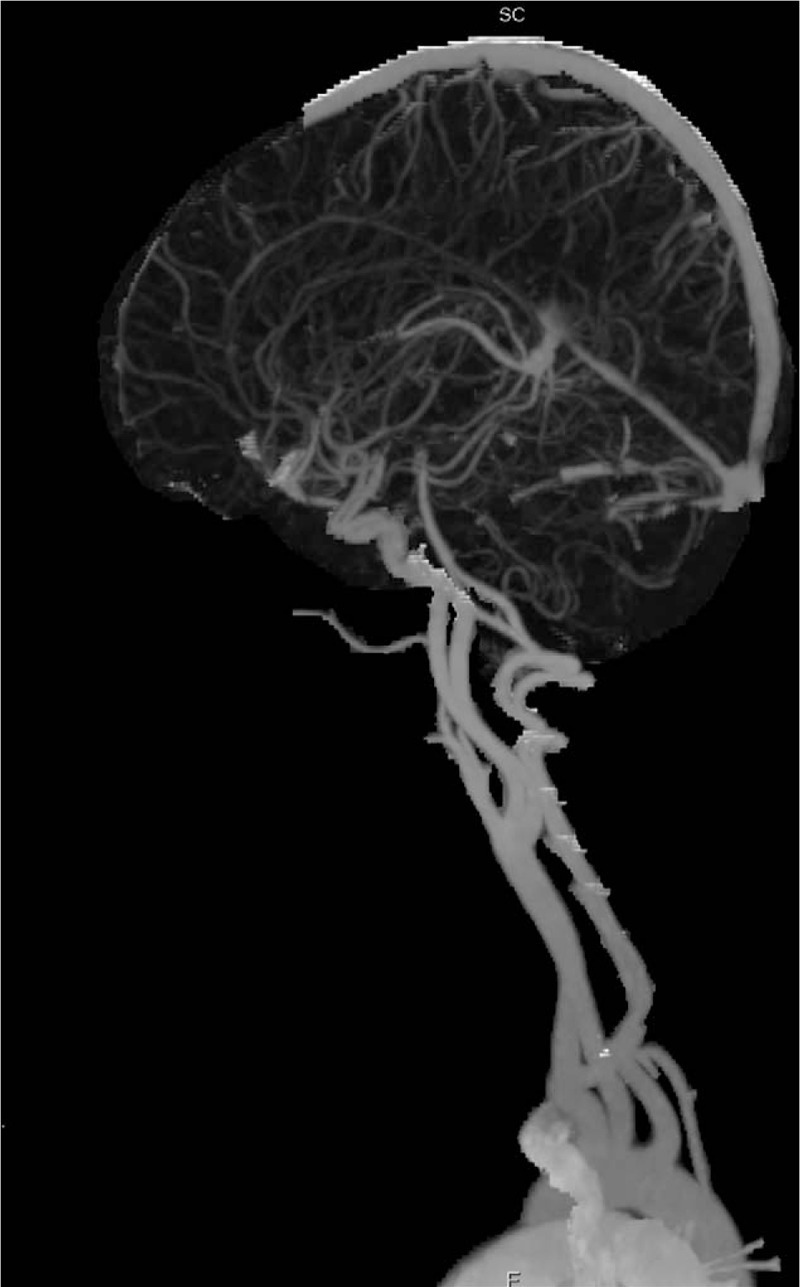
Research group: the same child, male, 6 years and 5 months, MIP images of the head and neck vessels. It can be seen from the figure that the head and neck arteries, the superior sagittal sinus and the straight sinus are well imaged.

Subjective evaluation of image quality of arteries: There were 47 cases of 4 points and 3 cases of 3 points in the control group and 34 cases of 4 points and 16 cases of 3 points in the research group. Subjective evaluation of image quality of veins: There were 47 cases of 4 points and 3 cases of 3 points in the control group and 5 cases of 4 points, 42 cases of 3 points and 3 cases of 2 points in the research group. The subjective evaluation score of the research group was lower than that of the control group (*P* < .05, the difference was statistically significant); the subjective evaluation score of vein images was lower in the research group than in the control group (*P* < .05, the difference was statistically significant) (Table 3, Figs. 6–8).

**Table 3:**
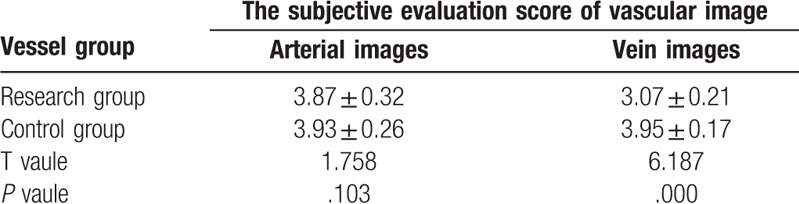
Subjective evaluation results of image quality of arteries and veins in children (n = 50, 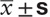).

### 4.3 Comparison of radiation dose and contrast agent dose in children

The effective radiation dose of X-rays to children in the research group was lower than that of the control group by approximately 51%, and the contrast agent dose that injected to the children in the research group was lower than that of the control group by approximately 44% (Table 4).

**Table 4:**
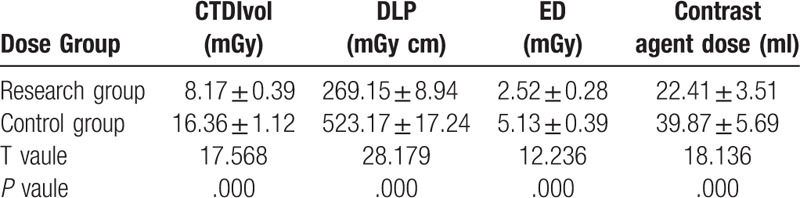
Results of X-ray dose and injection contrast dose in children (n = 50, 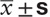).

## 5 Discussion

In this study, the contrast agent bolus with a diluted concentration of 20% was first injected, and then the undiluted contrast agent bolus was injected. By this method, we could scan the head and neck 1 time and clearly display images of the arteries and veins. The purpose of the first, diluted contrast agent bolus with a concentration of 20% is to develop the head and neck veins. The purpose of the following injection of an undiluted contrast agent bolus is to develop the head and neck arteries. The threshold of triggering the CT scan was set to be relatively high, at 180, making the contrast agent bolus with a concentration of 20% fail to reach the threshold, to avoid false triggering of the scan. When the CT value at the monitoring site reaches the trigger threshold, scanning is started. The first injected contrast agent bolus with a concentration of 20% began to fill the head and neck veins, and the latter, undiluted contrast agent bolus began to fill the head and neck arteries so that 1 CT scan could simultaneously develop the arteries and veins of the head and neck. The agents that fill the arteries are undiluted contrast agents, and the vascular enhanced CT value is relatively high, which is highlighted in development. The agents that filled the veins are contrast agents with a concentration of 20%, and the vascular enhanced CT value is relatively low, which shows relatively shallow development.

The doses of the contrast agent injected into the 2 groups of children were different. All the contrast agents in the control group were undiluted contrast agents. For the contrast agent bolus in the research group, half of the contrast agent was diluted to a concentration of 20% to fill the head and neck veins, and half of the contrast agent was undiluted to fill the head and neck arteries. Therefore, the total dose of contrast agent injected into the children in the research group was lower than that of the control group, which reduced the burden of renal filtration in the children.

In the evaluation of vascular image quality, the subjective evaluation scores in the research group and the control group all reached 3 points or more. This is because the 2 groups of children had the same contrast agent concentration in the arterial vessels during the CT scan, all of which were from undiluted contrast agent injection. In the objective evaluation of arterial images in the research group, the enhanced CT value of the arteries at each site was lower than that of the control group, and the subjective evaluation scores of the vascular images were lower than those of the control group. Although the concentrations of the contrast agent that filled in the arteries were the same in the 2 groups, both of which were from the undiluted contrast agents, the dose of the undiluted contrast agent in the research group was only half of that in the control group. In the objective evaluation of venous images in the research group, the enhanced CT value of the arteries at each site was lower than that of the control group, and the subjective evaluation scores were lower than those of the control group. For the CT scanning of children in the 2 groups, the agent that filled the veins of children in the control group was an undiluted contrast agent, while the agent that filled the veins of children in the research group was a contrast agent with a concentration of 20% due to its injection before scanning time. The later, undiluted contrast agent filled the arteries and did not reach the veins.

The dose of scanning radiation in the control group was nearly double that of the research group because children in the control group had to undergo 2 scans to complete the head and neck arterial and venous phases. In the conventional method of contrast agent bolus injection, the contrast agent cannot fully fill the arteries and veins at the same time during scanning. In the same period, if the arteries are filled well, then the veins are filled poorly; if the filling of veins is good, then the filling of arteries is poor. However, for children in the research group, only 1 scan was needed to scan the head and neck arteries and veins, and the arteries and veins were well filled with the contrast agents at the same time.

The limitation of this study is that the subjects in this study were all children aged 6 to 7 years. Children of other ages were not included in the study and will be examined in future studies. The present injection method of a contrast agent bolus can only be used in head and neck CT arteriovenous angiography. It cannot be applied to CT angiography in other parts. This is because the blood vessels in the head and neck have unidirectional blood flow and relatively fast blood flow velocity, and the contrast agent fills the arteries and veins relatively quickly. When the undiluted contrast agent fills the head and neck arteries, the contrast agent with a diluted concentration of 20% fills the head and neck veins.

In conclusion, the method of first bolus injection of 20% diluted contrast agent and later bolus injection of undiluted contrast agent can clearly demonstrate the head and neck arteries and veins with 1-time CT scanning, which greatly reduces the X-ray dose and contrast agent dose received by the children. It has great clinical significance and is worth promoting.

## Author contributions

Experimental design, project management
